# Brain glucose metabolism and nigrostriatal degeneration in isolated rapid eye movement sleep behaviour disorder

**DOI:** 10.1093/braincomms/fcad021

**Published:** 2023-02-02

**Authors:** Patricia Diaz-Galvan, Toji Miyagawa, Scott A Przybelski, Timothy G Lesnick, Matthew L Senjem, Clifford R Jack, Leah K Forsberg, Hoon-Ki Min, Erik K St. Louis, Rodolfo Savica, Julie A Fields, Eduardo E Benarroch, Val Lowe, Ronald C Petersen, Bradley F Boeve, Kejal Kantarci

**Affiliations:** Department of Radiology, Mayo Clinic, Rochester, MN 55905, USA; Department of Neurology, Mayo Clinic, Rochester, MN 55905, USA; Department of Quantitative Health Science, Mayo Clinic, Rochester, MN 55905, USA; Department of Quantitative Health Science, Mayo Clinic, Rochester, MN 55905, USA; Department of Information Technology, Mayo Clinic, Rochester, MN 55905, USA; Department of Radiology, Mayo Clinic, Rochester, MN 55905, USA; Department of Neurology, Mayo Clinic, Rochester, MN 55905, USA; Department of Radiology, Mayo Clinic, Rochester, MN 55905, USA; Department of Neurology, Mayo Clinic, Rochester, MN 55905, USA; Department of Neurology, Mayo Clinic, Rochester, MN 55905, USA; Department of Psychiatry and Psychology, Mayo Clinic, Rochester, MN 55905, USA; Department of Neurology, Mayo Clinic, Rochester, MN 55905, USA; Department of Radiology, Mayo Clinic, Rochester, MN 55905, USA; Department of Neurology, Mayo Clinic, Rochester, MN 55905, USA; Department of Neurology, Mayo Clinic, Rochester, MN 55905, USA; Department of Radiology, Mayo Clinic, Rochester, MN 55905, USA

**Keywords:** isolated REM sleep behaviour disorder, FDG, Lewy bodies disease, PET, SPECT

## Abstract

Alterations of cerebral glucose metabolism can be detected in patients with isolated rapid eye movement sleep behaviour disorder, a prodromal feature of neurodegenerative diseases with α-synuclein pathology. However, metabolic characteristics that determine clinical progression in isolated rapid eye movement sleep behaviour disorder and their association with other biomarkers need to be elucidated. We investigated the pattern of cerebral glucose metabolism on ^18^F-fluorodeoxyglucose PET in patients with isolated rapid eye movement sleep behaviour disorder, differentiating between those who clinically progressed and those who remained stable over time. Second, we studied the association between ^18^F-fluorodeoxyglucose PET and lower dopamine transporter availability in the putamen, another hallmark of synucleinopathies. Patients with isolated rapid eye movement sleep behaviour disorder from the Mayo Clinic Alzheimer’s Disease Research Center and Center for Sleep Medicine (*n* = 22) and age-and sex-matched clinically unimpaired controls (clinically unimpaired; *n* = 44) from the Mayo Clinic Study of Aging were included. All participants underwent ^18^F-fluorodeoxyglucose PET and dopamine transporter imaging with iodine 123-radiolabeled 2β-carbomethoxy-3β-(4-iodophenyl)-N-(3-fluoropropyl) nortropane on single-photon emission computerized tomography. A subset of patients with isolated rapid eye movement sleep behaviour disorder with follow-up evaluations (*n* = 17) was classified as isolated rapid eye movement sleep behaviour disorder progressors (*n* = 7) if they developed mild cognitive impairment or Parkinson’s disease; or isolated rapid eye movement sleep behaviour disorder stables (*n* = 10) if they remained with a diagnosis of isolated rapid eye movement sleep behaviour disorder with no cognitive impairment. Glucose metabolic abnormalities in isolated rapid eye movement sleep behaviour disorder were determined by comparing atlas-based regional ^18^F-fluorodeoxyglucose PET uptake between isolated rapid eye movement sleep behaviour disorder and clinically unimpaired. Associations between ^18^F-fluorodeoxyglucose PET and dopamine transporter availability in the putamen were analyzed with Pearson’s correlation within the nigrostriatal pathway structures and with voxel-based analysis in the cortex. Patients with isolated rapid eye movement sleep behaviour disorder had lower glucose metabolism in the substantia nigra, retrosplenial cortex, angular cortex, and thalamus, and higher metabolism in the amygdala and entorhinal cortex compared with clinically unimpaired. Patients with isolated rapid eye movement sleep behaviour disorder who clinically progressed over time were characterized by higher glucose metabolism in the amygdala and entorhinal cortex, and lower glucose metabolism in the cerebellum compared with clinically unimpaired. Lower dopamine transporter availability in the putamen was associated with higher glucose metabolism in the pallidum within the nigrostriatal pathway; and with higher ^18^F-fluorodeoxyglucose uptake in the amygdala, insula, and temporal pole on a voxel-based analysis, although these associations did not survive after correcting for multiple comparisons. Our findings suggest that cerebral glucose metabolism in isolated rapid eye movement sleep behaviour disorder is characterized by hypometabolism in regions frequently affected during the prodromal stage of synucleinopathies, potentially reflecting synaptic dysfunction. Hypermetabolism is also seen in isolated rapid eye movement sleep behaviour disorder, suggesting that synaptic metabolic disruptions may be leading to a lack of inhibition, compensatory mechanisms, or microglial activation, especially in regions associated with nigrostriatal degeneration.

## Teaser

Diaz-Galvan *et al*. report a pattern of hypo- and hyper-cerebral glucose metabolism in patients with isolated rapid eye movement sleep behaviour disorder. Hypermetabolism in certain regions was associated with nigrostriatal degeneration. This pattern also differed between patients with isolated rapid eye movement sleep behaviour disorder who clinically progressed and those who remain stable over time.

## Graphical Abstract

**Graphical Abstract:**
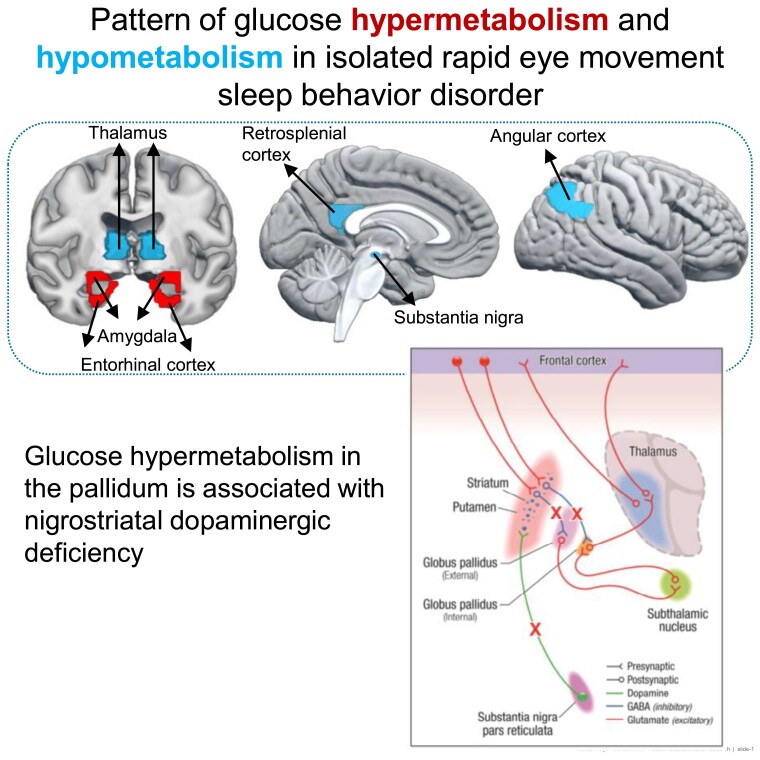

## Introduction

Rapid eye movement (REM) sleep behaviour disorder (RBD) is a parasomnia characterized by the loss of normal muscle atonia, enacting dreaming, and abnormal motor and vocal behaviour during REM sleep.^1,2^ When RBD symptoms occur in the absence of cognitive impairment, it is defined as the isolated form of RBD (iRBD).^3,4^ This condition of iRBD has been recognized as the earliest stage of progressive neurodegenerative diseases with α-synuclein pathologies, such as Parkinson’s disease (PD), dementia with Lewy bodies (DLB), or multiple system atrophy (MSA).^3,5–7^ In consequence, iRBD patients represent a suitable group to study the prodromal stage of synucleinopathies and would be an ideal target population for disease-modification trials.^8^ Biomarkers that can reliably identify iRBD patients at a higher risk of imminent clinical progression are needed for the enrichment of potential clinical trials.^9,10^

Molecular imaging, such as ^18^F-fluorodeoxyglucose (FDG) PET, is a powerful technology for understanding the metabolic alterations in neurodegenerative diseases.^11,12^ For example, patients with DLB are characterized by an FDG PET pattern of hypometabolism in parieto-occipital regions and a relatively preserved posterior cingulate metabolism, known as the cingulate island sign (CIS).^13^ In PD, FDG PET can detect the pattern of hypermetabolism in the pallidum, thalamus, pons, and motor cortical areas, as well as hypometabolism in the parietal and frontal cortical regions.^14,15^ Interestingly, these FDG PET findings are present even at the prodromal stages of the diseases, such as mild cognitive impairment with Lewy bodies (MCI-LB)^16,17^ or PD-MCI,^18,19^ and can predict clinical phenoconversion.^16^ In iRBD, the metabolic pattern is characterized by hypermetabolism in the cerebellum, brainstem, thalamus, sensorimotor cortex, and hippocampus, and by hypometabolism in the middle cingulate, parietal, temporal, and occipital cortices.^20,21^ However, further research is still needed to detect the metabolic characteristics that determine clinical progression in patients with iRBD.

Furthermore, a combination of biomarkers that are sensitive to different aspects of the disease processes may help understand the biological underpinnings of iRBD and can improve diagnostic and prognostic sensitivities of the biomarkers.^10^ Along with FDG PET, another imaging biomarker studied in iRBD is ^123^I-FP-CIT SPECT, which is sensitive to the dopaminergic abnormalities in the striatum associated with nigrostriatal degeneration, a hallmark of PD and DLB.^22,23^ In iRBD, ^123^I-FP-CIT SPECT imaging has identified dopaminergic abnormalities, which represent a reliable marker of progression.^24,25^ However, the interplay between FDG PET and ^123^I-FP-CIT SPECT findings still needs to be elucidated in iRBD, which may lead to a better understanding of the mechanisms of neurodegeneration.

Therefore, in this study we had three objectives: (i) to identify a metabolic signature of iRBD by comparing FDG PET in patients with iRBD with a clinically unimpaired (CU) group of controls; (ii) to investigate FDG PET findings in iRBD patients who developed cognitive impairment and/or PD compared with those who remained stable; and (iii) to study the association between FDG PET with nigrostriatal neurodegeneration on ^123^I-FP-CIT SPECT in iRBD.

## Materials and methods

### Participants

This study included patients with iRBD (*n* = 22) who were enrolled in the Mayo Clinic Alzheimer’s Disease Research Center (ADRC) and Center for Sleep Medicine; eight were also co-enrolled in the North American Prodromal Synucleinopathy (NAPS) Consortium. All patients underwent FDG PET and DaTscan imaging between 2013 and 2021. Diagnosis of iRBD was made according to the International Classification of Sleep Disorders-second edition (ICSD-II) criteria.^26^ These criteria required a clinical history of dream enactment behaviour. All patients underwent a polysomnographic recording which documented REM sleep without atonia, the electrophysiologic substrate for RBD. We excluded patients with psychiatric and/or neurological comorbidity, the presence of any other sleep disorder, and the use of medication, and/or substance abuse that may better explain the RBD symptoms. Clinically unimpaired participants (CU; *n* = 44) without cognitive, motor, or sleep disorders were also included as the control group and matched 2:1 to the iRBD patients on age, sex, and length of follow-up. The clinically unimpaired group was selected from the Mayo Clinic Study of Aging (MCSA), and participants were characterized as clinically unimpaired by a consensus of neurologists and neuropsychologists after a comprehensive in-person evaluation.^27^

A subset of iRBD patients (*n* = 17) was also followed over a range of 2 to 6 years [mean = 4.4 years, standard deviation (sd) = 2.0] with clinical evaluations performed at approximately annual intervals. We classified the patients into iRBD progressors (*n* = 7) if they developed cognitive impairment and/or PD, or as iRBD stables (*n* = 10) if their cognitive status remained unimpaired and no PD evolved throughout the follow-up. Among the iRBD progressors, six progressed to MCI-LB and only one to PD. Diagnoses of MCI-LB and PD were established by a team of neurologists according to published criteria after an in-person examination that included a comprehensive neuropsychological assessment.^28,29^ This subset of iRBD patients with follow-up (*n* = 17) were also age-, sex-, and follow-up time-matched to a subset of clinical unimpaired controls from the baseline control cohort (2:1 matching, *n* = 34).

Clinical information was obtained through a neurological interview and examination, and a neuropsychological assessment. The Mini-Mental State Examination (MMSE)^30^ and Clinical Dementia Rating Sum of Boxes (CDR-SOB)^31^ were used to assess the global cognitive status. Clinical features of DLB were assessed as described in previous reports from the DLB cohorts, such as in Choudhury *et al*. (2021).^32^ Briefly, parkinsonism was determined in the neurological examination as having at least two of the four cardinal features: tremor, rigidity, bradykinesia, and postural instability. The severity of parkinsonism was quantified with the Unified Parkinson’s Disease Rating Scale Part III (UPDRS-III).^33^ Visual hallucinations were considered to be present when they were fully formed, not restricted to a single episode, and not related to another medical issue, dementia, or treatment. The presence of fluctuations was determined by a score of 3 or 4 on the four-item Mayo Fluctuations Scale.^34^

The study was approved by the Mayo Clinic Institutional Review Board and informed consent was obtained from all participants.

### MRI, FDG PET, and ^123^I-FP-CIT SPECT acquisitions

MRI exams were performed at 3 Tesla. A 3D high-resolution magnetization prepared rapid gradient echo (MPRAGE; TR/TE/TI = 7/3/900 ms; flip angle = 8 degrees) with approximately 1 mm^3^ of the resolution was performed for anatomical segmentation and labelling of the FDG PET images. PET imaging was performed with PET/CT scanners (DRX; GE Healthcare; Siemens) operating in 3D mode. Patients were injected with an average of 348 MBq of ^18^F-FDG (range, 270–556 MBq). After a 30-min uptake period, we obtained four 3.75-minute dynamic frames. A CT image was obtained for attenuation correction. DaTscan was performed at a ^123^I-FP-CIT SPECT (GE Healthcare, Chicago, IL) according to a previously published protocol.^35^ In brief, at least 1 h before the injection of ^123^I-ioflupane, a 100 mg Lugol solution was given, and then the recommended ^123^I-ioflupane dose of 111–185 MBq (3–5 mCi) was slowly administered intravenously. SPECT imaging occurred 3–6 h after injection. GE D670/D630 SPECT systems with ultrahigh-resolution fan beam collimators and an energy setting of 159 keV 20% windows were used on all patients. Data were reconstructed with an ordered subset expectation maximization method; the planar images were prefiltered with a Butterworth filter (power 10, cutoff 0.6 cycles/cm). No attenuation correction was used. Projection images were used for the quantitative analysis.

### FDG PET analysis

FDG PET images were analyzed using an automated image analysis pipeline on SPM12. The pipeline consists of the following steps: (i) registration of the PET image volumes of each participant to their own T1-weighted MRI using 6 degrees of freedom affine registration with mutual information cost function; (ii) atlas-based segmentation and parcellation of FDG images into the region of interest (ROI) in each participant’s T1-weighted MRI space. We used the Mayo Clinic Adult Lifespan template (MCALT) for the parcellation of 49 grey matter ROIs,^36^ and DISTAL atlas for the substantia nigra;^37^ (iii) grey matter plus white matter sharpening to remove the effects of atrophy on regional FDG uptake; (iv) and calculation of the FDG standardized uptake value ratio (SUVr) in each voxel and ROIs. We used the median value of the pons uptake as the internal reference.

### ^123^I-FP-CIT SPECT analysis

Semiquantitative calculations of putaminal ^123^I-FP-CIT uptake on SPECT were performed by DaTQUANT™ software, Version 2 (GE Healthcare). The volumes of interest (VOIs) of DaTQUANT of fixed size were semi-automatically placed over the right and left putamen in the transaxial slice showing the most intense tracer uptake. Another VOI was placed over the occipital lobe representing the cortical background. The software automatically placed the same VOIs in the adjacent previous and following slices such that data from three contiguous slices were used. Then, the left and right striatum-to-background ratio (SBR) of the putamen was automatically calculated. Each SBR was classified as minimum, maximum, and average for raking purposes. Minimum SBR corresponds to the lowest value, maximum SBR corresponds to the highest value, and average SBR corresponds to the average between maximum and minimum SBRs. For this study, we used the average SBRs of the left and right putamen. We chose to study putamen SBR because we have previously shown that DaTQUANT scores from the putamen have the best discrimination between patients with autopsy-confirmed LB disease and those without LB disease.^35^

### Statistical analysis

Clinical and demographic characteristics were reported for the CU participants and iRBD patients as means and standard deviations for continuous variables, and counts (%) for categorical variables. Pairwise characteristic comparisons between CU and iRBD patients in the cross-sectional and longitudinal samples were done using conditional logistic regressions that account for the matching. The characteristics comparing iRBD stables and iRBD progressors were done using *t-*tests for continuous variables or *X*^2^ tests for categorical variables. The area under the receiver operation curves (*AUROC*) was calculated to measure the ability of FDG SUVr in each atlas-based ROI (50 ROIs) to distinguish iRBD patients from CU controls in the whole sample, or the subgroups of iRBD stables and iRBD progressors from CU in the subset with follow-up data. *AUROC*s were generated using a weighted two-stage parameter estimation approach accounting for matching on age, sex, and length of follow-up.^38^ ROI analysis using weighted *AUROC* for FDG SUVr was not corrected for multiple comparisons in order not to inflate the probability of Type II error. Unadjusted Pearson correlations were used for the associations of RBD onset age and RBD duration with FDG PET. Finally, we approached the study of the association between putamen ^123^I-FP-CIT SPECT and FDG PET in two ways: (i) we investigated the relationship between the putamen ^123^I-FP-CIT uptake and FDG SUVr in the nigrostriatal pathway nuclei using age-adjusted Pearson correlations and (ii) we investigated the putamen ^123^I-FP-CIT uptake and cortical FDG SUVr using a voxel-based approach. Voxel-based analyses were conducted using a regression model including putamen ^123^I-FP-CIT SPECT as a predictor and age as a covariate. All the voxel-based analyses were performed within the general linear model (GLM) framework of SPM12, and statistical maps were displayed at a significance of *P* < 0.001.

### Data availability

The data that support the findings of this study are available from the corresponding author upon reasonable request.

## Results

### Cohort characteristics

The baseline characteristics of the entire cohort are displayed in Table 1. Most patients with iRBD were men (*n* = 17; 77%) and the mean (*SD*) age was 65.4 (8.0) years. The CU group was similar to iRBD on age and sex by design. CU and iRBD groups did not differ on MMSE and CDR-SOB, consistent with the expected cognitive profile of iRBD. The duration of iRBD ranged from one year up to 25 years. A small proportion of iRBD patients showed additional core clinical features of DLB (*n* = 5; 20%), such as mild parkinsonism (*n* = 2; 1 progressor), visual hallucinations (*n* = 1 progressor), and cognitive fluctuations (*n* = 1 progressor).

**Table 1:** Baseline characteristics of participants

	CU *n* = 44	iRBD *n* = 22	*P*-value
Age, years	64.9 (7.5) [51.4, 83.0]	65.4 (8.0) [51.7, 83.3]	0.20
Males, no. (%)	34 (77%)	17 (77%)	1.00
APOE, no. (%)	12 (27%)	5 (28%)	0.84
Education, years	15.3 (2.2) [12, 20]	16.3 (2.6) [12, 20]	0.13
MMSE	28.8 (0.7) [27, 30]	28.7 (1.3) [26, 30]	0.73
CDR-SOB	0.0 (0.1) [0.0, 0.5]	0.2 (0.4) [0.0, 1.0]	0.091
Follow-up, years	3.2 (2.4) [0.0, 7.7]	3.4 (2.6) [0.0, 7.1]	0.29
DaTQuant Putamen	NA	1.82 (0.42) [0.56, 2.52]	—
DaTQuant Caudate	NA	2.18 (0.44) [0.77, 2.73]	—
Parkinsonism, no. (%)	NA	2 (10%)	—
Visual Hallucinations, no. (%)	NA	1 (5%)	—
Fluctuations, no. (%)	NA	1 (8%)	—
RBD Duration, years	NA	12.7 (12.5) [1, 41]	—
RBD Onset Age, years	NA	52.2 (17.1) [22, 77]	—

Values correspond to mean (SD) for the continuous variables and count (%) for the categorical variables. *P*-values for differences between groups come from conditional logistic models that account for the matching.

APOE, apolipoprotein E; CDR-SOB, Clinical Dementia Rating Sum Of Boxes; CU, clinically unimpaired; iRBD, idiopathic Rapid Eyes Movement (REM) sleep Behaviour Disorder; MMSE, Mini-Mental State Examination.

### FDG PET pattern in iRBD patients

**Figure 1:**
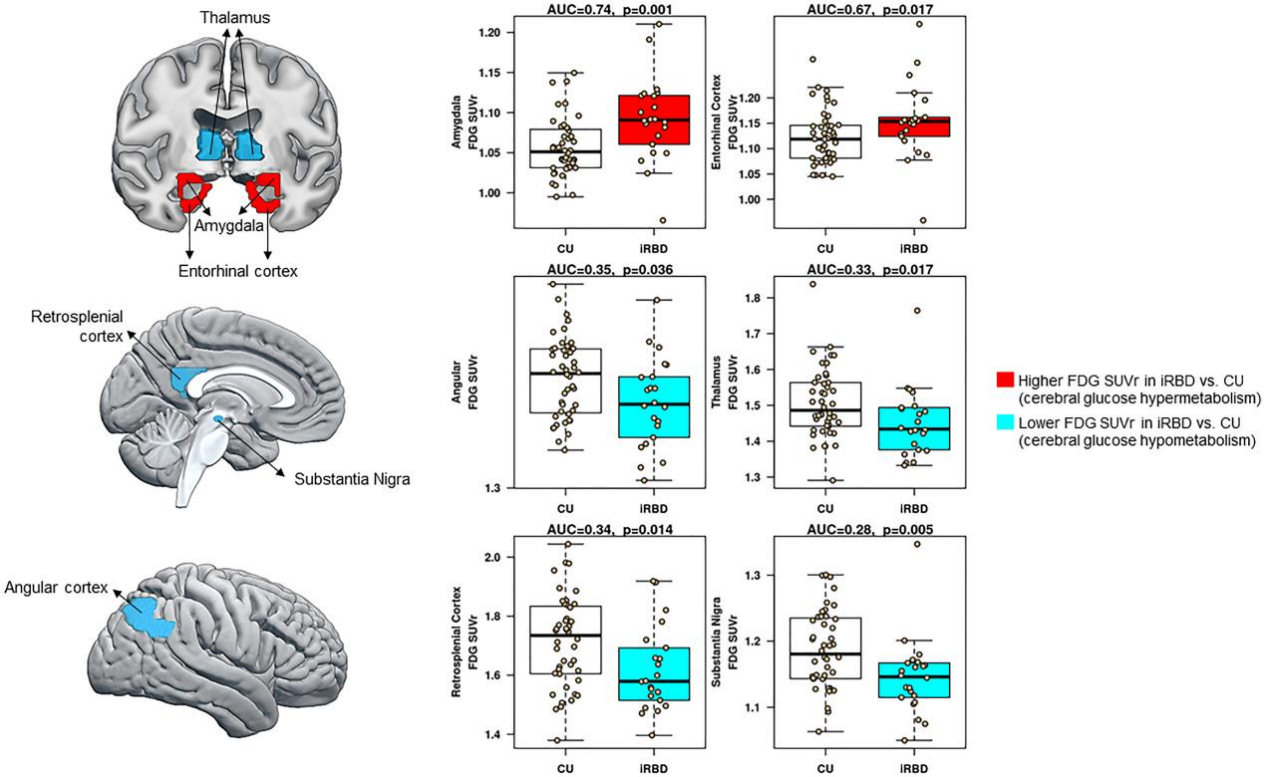
**Pattern of FDG PET glucose metabolism in iRBD.** We used weighted two-stage parameter estimation approach accounting for matching on age and sex to calculate area under the receiver operation curves (AUROC). AUROC tested the ability of FDG SUVr in each atlas-based ROI (50 ROIs) to distinguish iRBD patients from CU controls. FDG, ^18^F-fluorodeoxyglucose; SUVr, standardized uptake value ratio.

Atlas-based regional analyses were performed using the *AUROC* value, which measures the ability to distinguish iRBD and CU groups. Patients with CU were the reference group, such that *AUROC* values greater than 0.5 indicated higher FDG uptake in iRBD compared with CU. Conversely, *AUROC* values lower than 0.5 indicated lower FDG uptake in iRBD compared with CU group. Among the 50 atlas-based regions that we explored, results showed that patients with iRBD had lower FDG uptake in the substantia nigra (*AUROC* = 0.29, *P* = 0.005), restrosplenial cortex (*AUROC* = 0.31, *P* = 0.014), thalamus (*AUROC* = 0.32, *P* = 0.017), and angular gyrus (*AUROC* = 0.34, *P* = 0.036) compared with CU. On the contrary, patients with iRBD had higher FDG uptake in the amygdala (*AUROC* = 0.74, *P* = 0.001), and entorhinal cortex (*AUROC* = 0.68, *P* = 0.017) compared with CU. Figure 1 displays the iRBD FDG pattern together with boxplots showing the mean differences between CU and iRBD patients in these regions. *AUROC* plots from all the regions included in the analysis are summarized in Supplementary Fig. 1.

We also explored the association of regional FDG SUVr in the amygdala, entorhinal cortex, thalamus, retrosplenial cortex, angular cortex, and substantia nigra with disease duration and age of RBD onset. Only lower FDG PET SUVr in the thalamus was associated with a shorter duration of RBD (*r* = 0.48, *P* = 0.018) and later RBD onset (*r* = −0.51, *P* = 0.016). Lower FDG PET SUVr in the angular cortex was also associated with a shorter disease duration of RBD (*r* = 0.46, *P* = 0.030). Pearson correlations between symptom duration or age of RBD onset and FDG PET SUVr were not significant in the rest of the regions: the substantia nigra (disease duration: *r* = 0.22, *P* = 0.29; the age of onset: *r* = −0.08, *P* = 0.71), retrosplenial cortex (disease duration: *r* = −0.08, *P* = 0.72; the age of onset: *r* = −0.22, *P* = 0.32), entorhinal cortex (disease duration: *r* = 0.35, *P* = 0.11; the age of onset: *r* = −0.39, *P* = 0.07), and amygdala (disease duration: *r* = 0.34, *P* = 0.10; the age of onset: *r* = −0.33, *P* = 0.13).

### FDG PET findings in iRBD progressor and iRBD stable groups

A subset of 17 iRBD patients was followed up for a range of 2.4 to 6.4 years. Forty-one percent (*n* = 7) of the iRBD patients cognitively progressed and evolved to MCI-LB or developed PD, while 58% (*n* = 10) remained stable. Clinically, four iRBD patients had the core clinical features of DLB (parkinsonism, visual hallucinations, and/or cognitive fluctuation; Table 2). The age of iRBD onset was significantly higher for iRBD progressors than iRBD stable groups (*P* = 0.049).

**Table 2:** Baseline characteristics of patients with follow-up evaluations

	CU *n* = 34	iRBD *n* = 17	*P*-value^a^	iRBD Stable *n* = 10	iRBD Progressors *n* = 7	*P*-value^b^
Age, years	64.9 (7.4) [51.4, 83.0]	65.6 (8.0) [51.7, 83.3]	0.18	63.0 (7.2) [51.7, 72.9]	69.4 (8.1) [61.9, 83.3]	0.11
Males, no. (%)	24 (71%)	12 (71%)	1.00	7 (70%)	5 (71%)	0.95
APOE, no. (%)	11 (32%)	5 (29%)	0.84	4 (40%)	1 (14%)	0.25
Education, years	15.2 (2.2) [12, 20]	16.5 (2.7) [12, 20]	0.085	16.5 (2.5) [12, 20]	16.4 (3.2) [12, 20]	0.96
MMSE	28.8 (0.7) [27, 30]	28.7 (1.4) [26, 30]	0.73	29.1 (1.1) [27, 30]	28.1 (1.6) [26, 30]	0.16
CDR-SOB	0.0 (0.1) [0.0, 0.5]	0.1 (0.3) [0.0, 1.0]	0.31	0.0 (0.0) [0.0, 0.0]	0.3 (0.5) [0.0, 1.0]	0.080
Follow-up, years	4.1 (1.8) [0.0, 7.7]	4.4 (2.0) [2.1, 7.1]	0.29	4.1 (1.9) [2.1, 7.0]	4.9 (2.1) [2.1, 7.1]	0.39
DaTQuant Putamen	NA	1.76 (0.45) [0.56, 2.52]		1.90 (0.25) [1.46, 2.15]	1.55 (0.60) [0.56, 2.52]	0.11
DaTQuant Caudate	NA	2.11 (0.47) [0.77, 2.73]		2.23 (0.34) [1.73, 2.73]	1.95 (0.60) [0.77, 2.73]	0.25
Parkinsonism, no. (%)	NA	2 (12%)		1 (10%)	1 (14%)	0.79
Visual Hallucinations, no. (%)	NA	1 (6%)		0 (0%)	1 (14%)	0.22
Fluctuations, no. (%)	NA	1 (9%)		0 (0%)	1 (25%)	0.17
RBD Duration, years	NA	14.9 (13.4) [1, 41]		19.5 (15.0) [1, 41]	8.3 (7.6) [1, 20]	0.091
RBD Onset Age, years	NA	50.2 (18.4) [22, 77]		43.0 (19.2) [22, 70]	60.6 (11.9) [44, 77]	0.049

Values correspond to mean (SD) for the continuous variables and count (%) for the categorical variables.

APOE, apolipoprotein E; CDR-SOB, Clinical Dementia Rating Sum of Boxes; CU, clinically unimpaired; iRBD, idiopathic Rapid Eyes Movement (REM) sleep Behaviour Disorder; MMSE, Mini-Mental State Examination.

a *P*-values for differences between iRBD and CU come from conditional logistic models that account for the matching.

b *P*-values for differences between iRBD stables and progressors come from *t*-tests for the continuous variables or χ^2^ tests for the categorical variables. The *P*-values for differences between iRBD and groups come from conditional logistic models that account for the matching.

Regional FDG PET characteristics of iRBD progressor and iRBD stable groups were determined by using *AUROC* to distinguish each group of patients from an age- and sex-matched CU group. Compared with CU, iRBD progressors had higher FDG uptake in the entorhinal cortex (*AUROC* = 0.73, *P* = 0.020) and the amygdala (*AUROC* = 0.82, *P* = 0.031), and lower FDG uptake in the cerebellum (*AUROC* = 0.16, *P* = 0.012). Compared with CU, iRBD stables had higher FDG uptake in the amygdala (*AUROC* = 0.73, *P* = 0.049), cerebellum (*AUROC* = 0.74, *P* = 0.035), entorhinal cortex (*AUROC* = 0.74, *P* = 0.035), and fusiform gyrus (*AUROC* = 0.76, *P* = 0.022).

### Association of ^123^I-FP-CIT SPECT with FDG PET in iRBD

The investigation of the association between putamen ^123^I-FP-CIT SPECT uptake and FDG PET was approached in two ways: (i) the relationship between putamen ^123^I-FP-CIT uptake and FDG PET SUVr in the nigrostriatal pathway nuclei was investigated with age-adjusted Pearson correlations; (ii) putamen ^123^I-FP-CIT uptake and cortical FDG PET SUVr was investigated using a voxel-based analysis.

**Figure 2:**
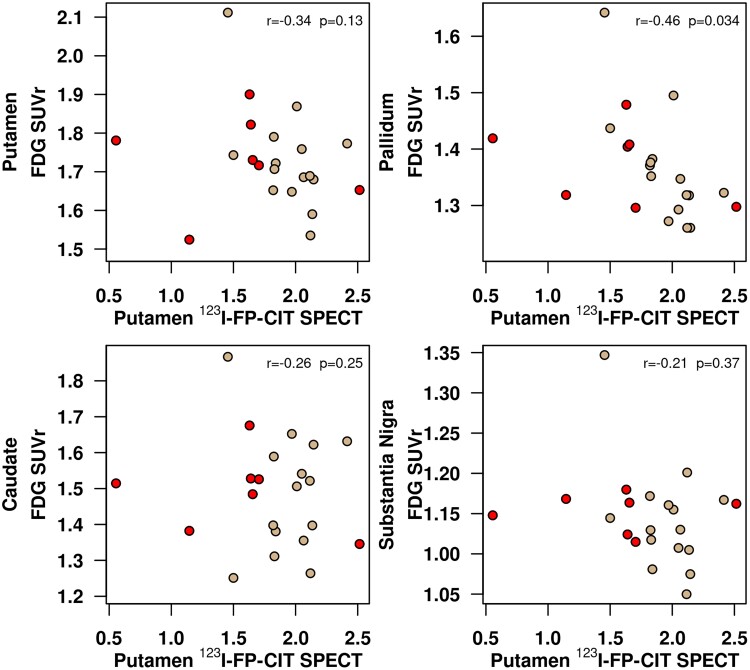
**Age-adjusted Pearson’s correlations between dopamine transporter availability in the putamen on ^123^I-FP-CIT SPECT (horizontal axis) and FDG uptake in each nigrostriatal nucleus (vertical axis).** Scatterplots display data of the entire cohort of iRBD patients (*n* = 22). Patients with iRBD who progressed over time are coloured in red. FDG, ^18^F-fluorodeoxyglucose; SUVr , standardized uptake value ratio.

Figure 2 shows the scatterplots for the associations of putamen ^123^I-FP-CIT uptake with FDG PET SUVr in the nigrostriatal pathway nuclei. Because there were no differences identified between the right and left nuclei, we display the results with the averaged FDG SUVr of both hemispheres with the average ^123^I-FP-CIT SBR. Lower putamen ^123^I-FP-CIT uptake was significantly associated with higher FDG SUVr in the pallidum (*Rho* = −0.46; *P* = 0.034***)***. The putamen^123^I-FP-CIT uptake was not significantly associated with FDG SUVr in the other nigrostriatal nuclei.

**Figure 3:**
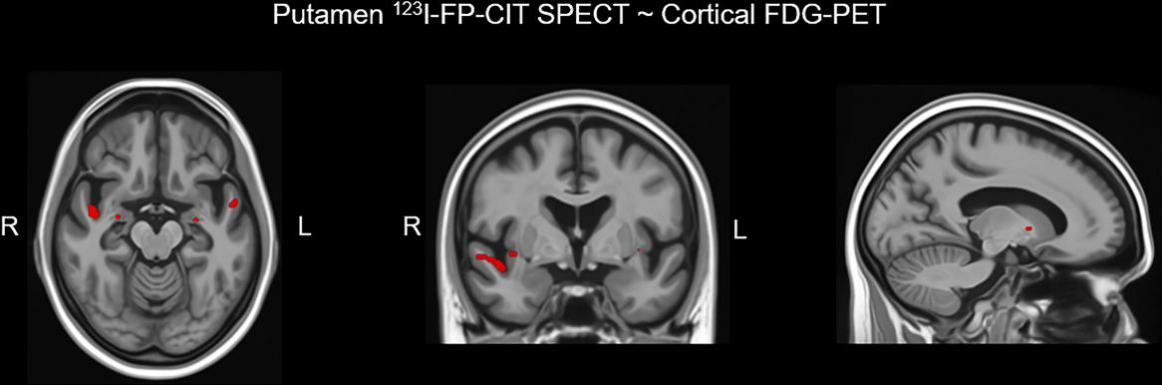
**Voxel-based analysis of the association between the dopamine transporter availability in the putamen on ^123^I-FP-CIT SPECT and FDG PET SUVr in cortex.** Maps of this association are displayed at the *P* < 0.001 level for the entire cohort of iRBD patients (*n* = 22). Colours towards red indicate higher FDG SUVr or hypermetabolism in association with lower dopamine transporter availability in the putamen. The voxel-based regional associations of putamen ^123^I-FP-CIT SPECT and FDG PET SUVr were corrected for multiple comparisons using family-wise error correction. Because there were no significant associations between putamen ^123^I-FP-CIT SPECT and FDG PET after correction for multiple comparisons, we display the uncorrected results.

Figure 3 shows the voxel-based associations between ^123^I-FP-CIT uptake and cortical FDG SUVr. Since there were no significant associations after correction for multiple associations, we display the uncorrected results (*P* < 0.001). Lower putamen ^123^I-FP-CIT uptake was associated bilaterally with higher FDG SUVr in the amygdala, insula, and superior temporal gyrus. Lower ^123^I-FP-CIT uptake in the putamen was also associated with higher FDG PET SUVr in the right pallidum (*P* < 0.001).

## Discussion

Our findings demonstrate that cerebral glucose metabolism in iRBD is characterized by both hypometabolic and hypermetabolic patterns on FDG PET. The topographic pattern of hypometabolism involves the substantia nigra, the retrosplenial cortex, the thalamus, and the angular gyrus, which is consistent with FDG PET studies in MCI-LB.^16^ However, patients with iRBD also show hypermetabolism in the amygdala and the entorhinal cortex. Moreover, increased glucose metabolism in the amygdala and entorhinal cortex, together with decreased glucose metabolism in the cerebellum, characterized those patients who became cognitively impaired or developed PD during follow-up (i.e. iRBD progressors). The hypermetabolism observed in the amygdala was associated with the loss of dopaminergic activity in the putamen, measured by dopamine transporter availability on ^123^I-FP-CIT SPECT. This loss of dopaminergic activity in the putamen was also associated with glucose hypermetabolism in the pallidum, amygdala, insula, and superior temporal cortex. Altogether, our data indicate FDG PET hypometabolism in iRBD that may reflect synaptic dysfunction, but also hypermetabolism, which happens simultaneously and is in part associated with nigrostriatal dopaminergic deficiency and clinical progression.

In this study, we have shown that FDG alterations in iRBD are characterized by hypermetabolism in limbic structures. Regional hypermetabolism has been observed in previous studies in PD and iRBD, indicating increased glucose metabolism in the cerebellum, basal ganglia, and frontal cortical regions.^14,15,20,39,40^ Interestingly, we found increased glucose metabolism in the amygdala and entorhinal cortex also in those patients who progressed during follow-up. In synucleinopathies where RBD is frequent, such as PD or DLB, pathological changes are common in the basal forebrain and limbic structures including the amygdala.^41,42^ Furthermore, the amygdala is one of the anatomic structures implicated in the generation of REM sleep through both direct and indirect projections from the brainstem.^43,44^ However, little is known about the involvement of the amygdala and limbic system in RBD, so the mechanism underlying the observed metabolic hyperactivity is still unclear.

One explanation for the limbic hypermetabolism in iRBD might rely on the dysfunction of reciprocal connections between the amygdala and brain stem nuclei that generate and modulate REM sleep, such as coeruleus/subcoeruleus complex or raphe nuclei. A previous animal study identified REM-ON cells in the central nucleus of the amygdala.^45^ In normal conditions, REM-ON cells in the amygdala are inhibited by serotonin (and possibly GABA) input from the dorsal raphe nucleus in the brain stem. Therefore, the hyperexcitability of the amygdala observed in RBD might be the result of a lack of inhibitory input from different groups of cells in the brainstem. These groups of cells in the brain stem are amongst the earliest and most severely affected regions by Lewy body pathology.^46^

Another possible explanation for the limbic hypermetabolism in iRBD relies on compensatory mechanisms occurring as a response to initial neuronal damage during the early stages of neurodegenerative diseases. For example, in Alzheimer’s disease (AD), compensatory mechanisms have been documented over the years and comprise a wide spectrum of biological processes, including increased brain glucose metabolism in selective cortical areas (see Merlo *et al.*^47^ for a systematic review). In AD, the increase in glucose metabolism has been interpreted as a compensatory response that promotes resilience to pathology progression at early stages, delaying the conversion from MCI to dementia.^48–50^ However, as the disease progresses and the pathology load worsens, the hypermetabolism is followed by a decline in brain metabolism.^51^ From these data, we hypothesize that compensatory hypermetabolism might be happening in synucleinopathies when patients are diagnosed with iRBD as an early outcome of increasing *α*-synuclein burden, which begins to accumulate in the olfactory cortex and brain stem.^46^ However, it is unclear whether limbic hypermetabolism is an indication of progression to MCI or dementia in iRBD patients. In fact, our findings indicate that those iRBD patients who progressed over time (i.e. iRBD progressors) show hypermetabolism in the amygdala and the entorhinal cortex. On the contrary, those iRBD patients who remained stable over time (i.e. iRBD stables) did not show any hypermetabolic activity compared with controls; whether this stable group represents those with iRBD who are early in the course of synucleinopathy evolution and hence many years or decades from progressing to MCI and/or parkinsonism, or have a non-degenerative aetiology for the RBD, will require more longitudinal assessments. Previous works in AD have shown that higher regional brain metabolism is associated with higher tau deposition on PET and with episodic memory impairment in MCI,^52^ and that neuronal hyperexcitability may drive tau release, propagation, and spread at the initial phases of the disease.^53,54^ This phenomenon may also occur in other neurodegenerative diseases, such as Parkinson’s disease or dementia of Lewy bodies. Hence, it is possible that early hypermetabolism leads to an exacerbation of neuronal damage and represents a maladaptive, detrimental event, rather than a beneficial compensatory response.^55^

Finally, one of the biological events occurring during the earliest stages of neurodegenerative diseases is neuroinflammation,^56^ which activates microglial cells.^57–59^ Microglial cells hold an active role in neuronal function, providing structural support and modulating synaptic transmission.^60^ In neurodegenerative diseases, microglia show activation through overexpression of transmembrane triggering receptors expressed in myeloid cells 2 (TREM2).^61^ In AD, this activation of the microglia favours an anti-inflammatory phenotype in response to increasing amyloid load.^62^ Recently, Xiang *et al.*^63^ showed that FDG PET uptake is strongly influenced by microglial activity in patients with neurodegenerative diseases. Furthermore, their mouse model showed that elevated microglial FDG uptake correlated with increased expression of the 18-kDa translocator protein (TSPO) on PET, which has been consistently associated with neuroinflammation.^56^ Overall, their results suggest that microglia activation occurring at early stages of neurodegenerative diseases, most probably because of an inflammatory process, stimulates glucose uptake and thus drives the increase of FDG PET signal (i.e. hypermetabolism). In Lewy body disease, one of the earliest and most severe Lewy body pathologic changes in the cerebral gray matter occurs in the amygdala and entorhinal cortex.^46^ The increasing *α*-synuclein accumulation triggers neuroinflammation, which leads to microglial activation, which can potentially increase FDG uptake in these regions. In summary, there are multiple mechanisms that could explain the increased glucose metabolism in the amygdala and entorhinal cortex in iRBD, perhaps occurring independently or even co-existing. Further research is needed to elucidate these mechanisms and their contribution to iRBD progression.

Our findings also indicate a hypometabolic pattern characterized by lower FDG uptake in the substantia nigra, retrosplenial cortex, thalamus, and angular gyrus. Neurodegeneration of the substantia nigra in association with a decreased dopaminergic input to the striatum is a hallmark of Lewy body disease.^64^ A pattern of reduction of glucose metabolism in the substantia nigra has been reported previously in PD and MCI-LB, and it has additive value in distinguishing patients with prodromal dementia of Lewy bodies from those with prodromal AD.^16,65^ Current results provide evidence that metabolic alterations in the substantia nigra occur in iRBD as an early biomarker of Lewy body disease. More research is needed to elucidate if the hypometabolism in the substantia nigra is a feature of those patients who will develop parkinsonism. Considering the low proportion of iRBD patients with features of parkinsonism in our cohort (9%; two patients), the current study suggests that hypometabolism in the substantia nigra is a metabolic alteration that characterizes patients within the whole spectrum of Lewy body pathology. A consistent pattern of hypometabolism involving the occipital and parietal cortex has been observed in iRBD.^20,39,40^ In the current study, we also found hypometabolism, but it was constrained to the retrosplenial cortex and angular gyrus. The retrosplenial cortex is one of the few limbic regions activated during REM sleep.^66^ During REM sleep, the retrosplenial cortex is activated by glutamatergic neurons of the claustrum, which are involved in sleep by promoting slow wave activity.^67^ The activation of glutamatergic neurons in the retrosplenial cortex leads to the inhibition and control of several motor regions that control the muscle atonia during REM sleep. Therefore, the inactivity in the retrosplenial cortex in iRBD might be explained by the dysfunction of the REM sleep circuit itself because of a reduction of glutamatergic input from REM sleep centres in the brainstem. However, decreased metabolism in the retrosplenial cortex and angular gyrus could also be explained by the pathologic spreading of α-synuclein. Patients with DLB have shown greater hypometabolism involving retrosplenial cortex and angular gyrus, together with other posterior temporal, parietal, and occipital regions.^68^ This hypometabolic pattern is thought to be related to impaired cholinergic synaptic dysfunction as a result of the accumulation of α-synuclein in the basal forebrain,^69,70^ which occur relatively early in the progression of the LB disease (i.e. consistent with *Stage 4* in Braak’s α-synuclein brain pathology staging model).^46^

The thalamic hypometabolism observed in our iRBD patients is consistent with the findings in MCI-LB and DLB.^13,16,68^ On the contrary, studies in Parkinson’s disease and iRBD have found hypermetabolism in the thalamus, instead of hypometabolism.^20,71^ These conflicting findings may be explained by differences in study design. While most of the previous studies in iRBD have been focused on detecting the PD-related metabolic pattern,^39,72,73^ in the current study we followed an unsupervised method to describe overall metabolic alterations. Therefore, the metabolic pattern found in the current study might reflect glucose metabolic alterations in iRBD during the very early stages in the continuum of DLB instead of PD. In fact, the proportion of patients with parkinsonism was low (9%) and, among those who progressed over time, only one patient progressed to PD, while the rest received a diagnosis of MCI-LB (*n* = 6). Metabolic patterns found in iRBD patients may vary by different pathological pathways associated with DLB, PD, or MSA. More longitudinal studies with larger cohorts are needed to test these hypotheses. Following a sequence of metabolic events of hypermetabolism preceding hypometabolism,^51^ it is also possible that the thalamic hypometabolism observed in the current study reflects a later stage in iRBD. This is supported by the proportion of progressors (30%) who developed MCI-LB or Parkinson’s disease within an average of five years. This high proportion of progressors suggests that most of the patients in this study were already in a later stage of iRBD. A good example of this alteration in the metabolic pattern by disease progression is amygdalar hypometabolism observed in MCI-LB and dementia of Lewy bodies,^13,16^ but hypermetabolism observed in iRBD patients.

Hypometabolism was also found in the cerebellum, but only for iRBD patients who progressed to MCI-LB or Parkinson’s disease over time. Altered metabolic connectivity in the cerebellum is a common finding in patients with either PD or DLB, suggesting that the cerebellum is a vulnerable region to Lewy body pathology.^74^ However, the metabolic pattern in the cerebellum in iRBD is not fully understood. Contrary to our study, Meles *et al.*^20^ found increased metabolic activity in the cerebellum, while Carli *et al.*^75^ tested individual metabolic patterns in patients with iRBD and found that a high proportion of them had occipito-cerebellar hypometabolism. This heterogeneity may be explained again by the variation in iRBD patients at different stages of the iRBD pathophysiology. Furthermore, other neuroimaging studies in iRBD have also shown loss of grey matter volume and perfusion abnormalities in the cerebellum. While some studies include cohorts with a more recent diagnosis of iRBD, other cohorts may include patients who have more advanced diseases, such as the group of progressors in our cohort. On the one hand, this reduction in cerebellar metabolic activity in iRBD might be explained by the loss of neural tissue and perfusion observed in previous studies,^76^ and it may be due to a vulnerability of the cerebellum to Lewy body pathology. This cerebellar hypometabolism may also reflect a noradrenergic dysfunction due to the Lewy body pathology in the locus coeruleus. Locus coeruleus is affected by Lewy pathology during the earliest phases of the disease,^46,77^ which may reduce its noradrenergic connections to the cerebellum and facilitate hypometabolic alterations.^78^

**Figure 4:**
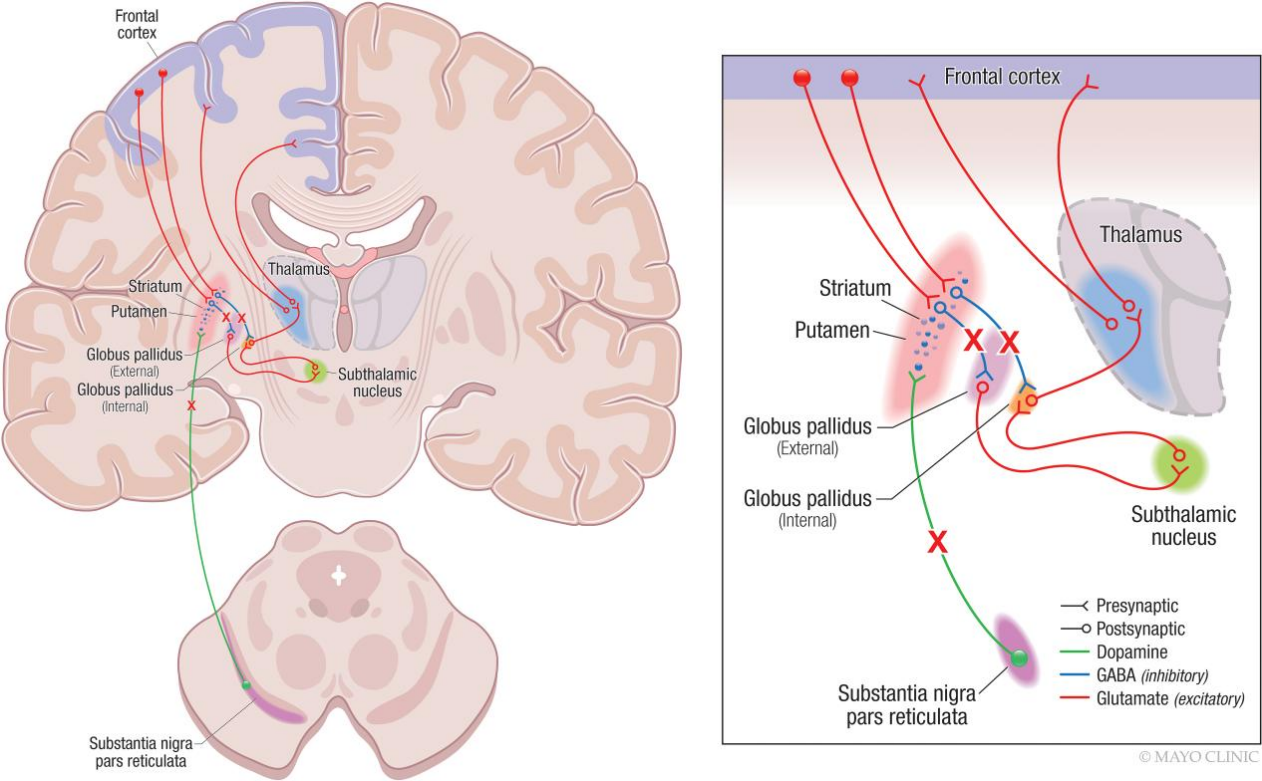
**Alterations of nigrostriatal pathway in iRBD.** Image on the right illustrates a neuroanatomic representation of the nigrostriatal pathway as well as cortico-striatal and cortico-thalamic connections. Image on the left illustrates a zoomed scheme of the nigrostriatal pathway. Filled circles represent neuron nuclei in the subtantia nigra and cortex from which the striatum receives dopamine and glutamine input respectively. Lines ending with a V shape represent presynaptic neurons in the corresponding tracks. Lines starting with a hollow circle represent postsynaptic neurons in the tracks. Crosses illustrate the tracks that are disrupted because of a degeneration of the susbtantia nigra in iRBD. Briefly, the substantia nigra pars compacta sends dopaminergic input to the putamen to modulate its activity (in green). The putamen has a direct and indirect inhibitory GABAergic connection with the pallidum. When the putamen receives dopamine from the substantia nigra, it activates the release of GABA to the pallidum, which inhibit its activity (in blue). If there is neurodegeneration of the substantia nigra, there is a lack of dopamine input to the putamen, which leads to a lack of inhibition of the pallidum. Consequently, the pallidum will be over-activated, showing hypermetabolism that can be detected on FDG PET.

Dopamine transporter abnormalities have been consistently identified in iRBD.^77,79–85^ Interestingly, dopamine transporter abnormalities have been associated with more severe RBD symptoms and an accelerated rate of progression to dementia.^86–88^ However, very little is known about the interplay between ^123^I-FP-CIT SPECT and FDG PET findings in iRBD. Only Meles *et al.*^73^ reported data from both modalities in the same study. They showed that the Parkinson’s disease-related FDG pattern can be identified in patients with iRBD compared with healthy individuals and that the expression of this pattern was higher in iRBD patients with abnormal dopamine transporter SPECT scan. Nonetheless, regional associations of both modalities still need to be elucidated. Within the nigrostriatal pathway, we found that decreases in dopamine transporter availability in the putamen were associated with glucose hypermetabolism in the pallidum. The hypermetabolism in the pallidum associated with the reduction of dopamine transporter activity in the putamen may reflect a disruption of the inhibitory input from the striatum to the pallidum, which occurs early in synucleinopathies.^89,90^Figure 4 displays a schematic representation of some of the excitatory and inhibitory connections within the nigrostriatal pathway. In a simplified manner, dopaminergic input from substantia nigra to putamen modulates part of the activity in the striatum, and thus, other components of the circuits. When there is neurodegeneration in the substantia nigra, the dopaminergic input to the putamen decreases and it leads to a lack of direct and indirect inhibitory input to the globus pallidus.^64^ We hypothesize that a lack of inhibitory input from the putamen to the pallidum due to the degeneration of the substantia nigra and nigrostriatal pathway connections most likely leads to hypermetabolism in the pallidum. However, other mechanisms could contribute to the hypermetabolic activity observed in the pallidum. For example, the substantia nigra has also direct connections to the pallidum, so it is possible that metabolic alterations in the pallidum are consequences of the dysfunction of direct dopaminergic innervation from the substantia nigra to the pallidum. Hypermetabolism in the pallidum might be also due to compensatory mechanisms or neuroinflammation. Further data are needed to test these hypotheses in future research. On a voxel-based analysis throughout the cortex, we also found that lower dopamine transporter availability in the putamen was associated with higher FDG uptake in the amygdala, the insula, and the superior temporal gyrus. The association of glucose hypermetabolism in these cortical regions with less dopamine transporter availability in the putamen might be explained by the dysfunction of some indirect connections between them. However, these significant associations might also be explained by the Lewy body-related pathological processes that are occurring simultaneously in the brain. Previous studies have already shown that pathological changes in prodromal DLB and DLB involve limbic structures such as the amygdala.^41,42^ Some metabolic and structural changes in the insula and lateral temporal cortex have been also reported even in patients with iRBD in association with lower performance in visuoperceptive and visuospatial functions.^91^ Further research is needed to understand whether and how iRBD-related metabolic alterations in the cortex are mediated by a nigrostriatal dopaminergic deficiency in conjunction with α-synuclein inclusions.

We acknowledge several limitations of our study. One limitation was a relatively small sample size, especially for those patients with follow-up data. Because the study cohort was very small for those who progressed to MCI-LB or PD, the FDG PET signatures found in the subgroup of iRBD progressors still need to be tested and corroborated in larger and independent cohorts, so we can determine their utility to predict clinical progression. In addition, our cohort comes from an ADRC and Center for Sleep Medicine, therefore movement disorders, such as PD, might be underrepresented as a possible endpoint of iRBD progression. Larger cohorts with a wide representation of these conditions and longer follow-up intervals are still needed in the study of biomarkers in iRBD. Another limitation was that most of the iRBD patients in our study were men. Although men are frequently over-represented in iRBD cohorts,^5^ the origin of this gender predominance may be motivated by both biological reasons and other explanations such as a women’s tendency not to seek medical attention for iRBD. Hence, the power to investigate differences in the FDG PET profile between men and women was limited in this study. Furthermore, our data are cross-sectional and cannot provide information on the temporal evolution of FDG PET pattern and its longitudinal association with dopamine biomarkers in iRBD. In consequence, we still cannot conclude on the cause-and-effect relationship between neurodegeneration and dopaminergic deficit in iRBD. Further investigations in larger cohorts and with a longitudinal design are needed to investigate the trajectory of metabolic alterations in iRBD together with its association with other biomarkers and disease progression.

The next challenge in the treatment of synucleinopathies is to test disease-modifying therapies in people with iRBD with the aim of slowing, or even preventing, the full manifestation of the Lewy body diseases. With this purpose, it is important to enrich target iRBD populations with biomarkers that improve our understanding of the biological alterations and neurodegeneration in synucleinopathies. This study provides data about glucose metabolic alterations that are indicative of both hypometabolism and hypermetabolism in iRBD. These data can guide clinical decisions on combined treatments that target both mechanisms. Another priority in future clinical trials is the combination of biomarkers that span multiple modalities. Our data on the association between FDG PET and ^123^I-FP-CIT SPECT validate previous studies on nigrostriatal dopaminergic degeneration and provide useful information on networks that are directly and indirectly affected in iRBD.

## Supplementary Material

fcad021_Supplementary_Data

## Abbreviations

ADRC =: Azheimer’s disease Research Center
AUROC =: area under the receiver operation curve
CIS =: cingulate island sign
CDR-SOB =: Clinical Dementia Rating Sum Of Boxes
CU =: clinically unimpaired
DLB =: dementia with Lewy bodies
FDG =: ^18^F-fluorodeoxyglucose
ICSD-II =: International Classification of Sleep Disorders-second edition
^123^I-FP-CIT =: ^123^I-N-ω-fluoropropyl-2β-carbomethoxy-3β-(4-iodophenyl)
iRBD =: isolated rapid eye movement behaviour disorder
LB =: Lewy body
MCALT =: Mayo Clinic adult lifespan template
MCI =: mild cognitive impairment
MCSA =: Mayo Clinic study of aging
MMSE =: Mini-Mental state examination
MPRAG =: magnetization prepared rapid gradient echo
MSA =: multiple system atrophy
NAPS =: North American Prodromal Synucleinopathy
PD =: Parkinson’s disease
REM =: rapid eye movement
ROI =: region of interest
SBR =: striatum-to-background ratio
SPECT =: single-photon emission computed tomography
SUVr =: standardized uptake value ratio
TE =: echo time
TI =: inversion time
TR =: repetition time
TREM2 =: transmembrane triggering receptor expressed in myeloid cells 2
TSPO =: 18-kDa translocator protein
UPDRS-III =: Unified Parkinson’s Disease Rating Scale Part III
VOI =: volume of interest.
