# Supplementary material for: Brain glucose metabolism and nigrostriatal degeneration in isolated rapid eye movement sleep behaviour disorder
Source: Brain Commun. 2023 Feb 2;5(1):fcad021. doi: 10.1093/braincomms/fcad021 (PMC9945851; doi:10.1093/braincomms/fcad021)
Supplement: fcad021_Supplementary_Data [file fcad021_supplementary_data.docx]

**
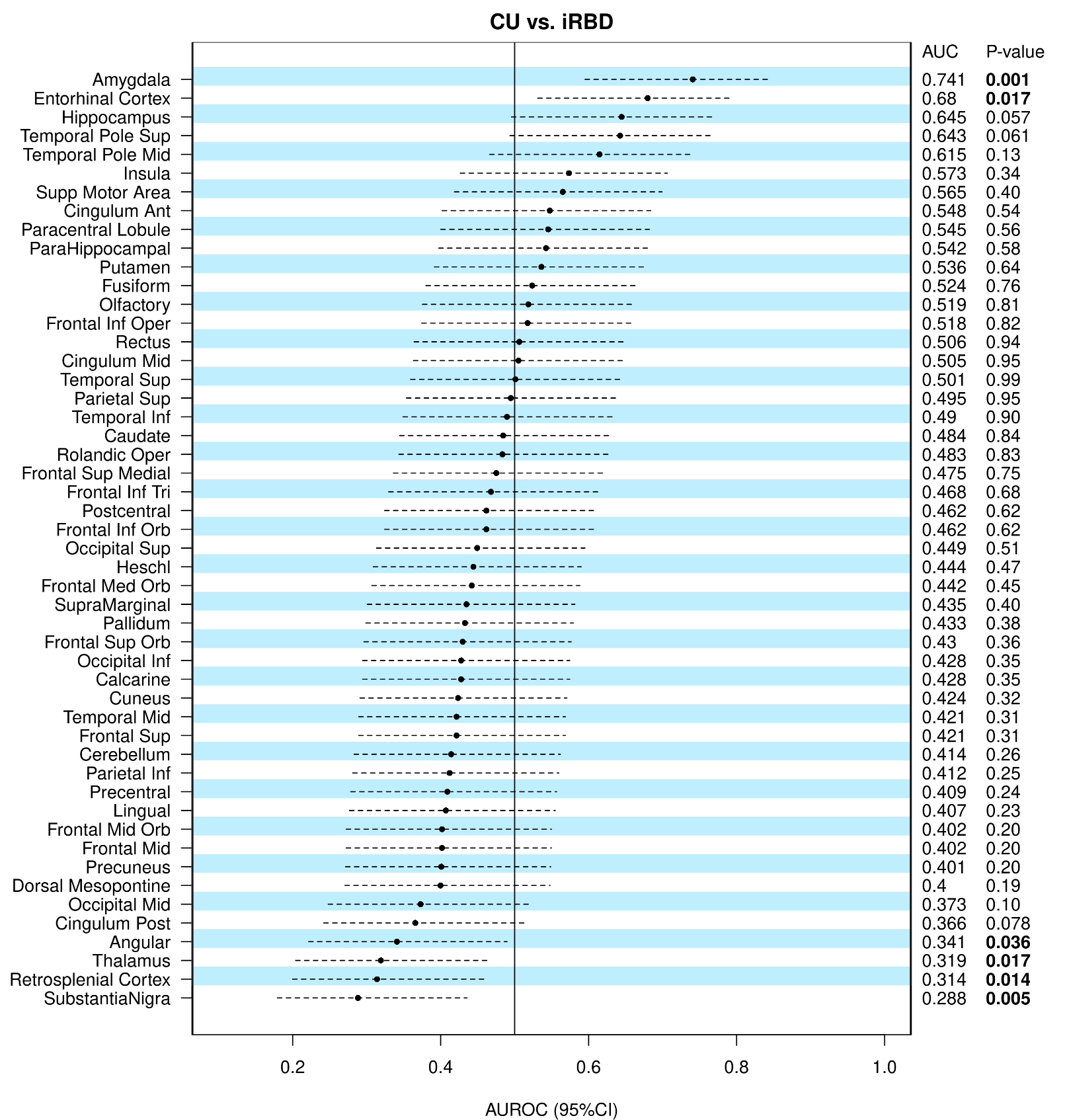

Supplementary Figure 1 - Forest plot demonstrating comparisons of atlas-based regional FDG SUVr between CU and iRBD groups.** Area under the receiver operation curves (*AUROC*) were calculated to measure the ability of FDG SUVr in each atlas-based ROI (50 ROIs) to distinguish iRBD patients from CU controls. Patients with CU were the reference group, such that *AUROC* values greater than 0.5 indicate higher FDG uptake in iRBD compared to CU; *AUROC* values lower than 0.5 indicate lower FDG uptake in iRBD compared to CU group. *AUROC* values and corresponding p-values are displayed in the forest plot for each ROI. Significant comparisons are indicated in bold text.
